# Metformin and Placental Ferroptosis in Gestational Diabetes: A Mechanistic Study

**DOI:** 10.1155/jdr/2226729

**Published:** 2026-06-03

**Authors:** Qiannan Lin, Xinyu Qin, Meihong Shen, Dandan Xia, Xiaorong Cao, Guangtong She, Huiyan Wang, Wenbo Zhou

**Affiliations:** ^1^ Department of Obstetrics and Gynecology, Changzhou Maternal and Child Health Care Hospital, Changzhou Medical Center, Nanjing Medical University, Changzhou, Jiangsu, China, njmu.edu.cn; ^2^ Department of Obstetrics, Taizhou Jiangyan Hospital of TCM, Nanjing University of Chinese Medicine, Taizhou, Jiangsu, China, njucm.edu.cn; ^3^ Medical Research Center, Changzhou Maternal and Child Health Care Hospital, Changzhou Medical Center, Nanjing Medical University, Changzhou, Jiangsu, China, njmu.edu.cn

**Keywords:** ferroptosis, gestational diabetes mellitus, metformin, trophoblasts

## Abstract

Gestational diabetes mellitus (GDM) is a major contributor to adverse pregnancy outcomes. Although the involvement of ferroptosis in GDM pathogenesis has been recognized, the precise mechanisms remain incompletely understood. This study is aimed at investigating the potential regulatory effects of metformin (Met) on ferroptosis in GDM. Patients with GDM were stratified into four groups: diet‐controlled (Group 1), nonmedicated (Group 2), Met‐treated (Group Met), insulin‐treated (Group Ins), alongside healthy controls (Group N). Placental trophoblasts and HTR‐8/SVneo cells were subjected to analysis using transmission electron microscopy (ultrastructure), Prussian blue staining (iron detection), CCK‐8 assay (proliferation), DCFH‐DA probe (reactive oxygen species [ROS]), and biochemical assays (Fe^2+^, glucose, insulin resistance, and reduced glutathione, GSH). TNF‐*α* and IL‐10 levels were measured via flow cytometry. The expression of ATF2, ACSL4, GPX4, and NRF2 was assessed by Western blot and quantitative PCR (qPCR). The ultrastructural characteristics of ferroptosis were observed in both placental trophoblasts and HTR‐8/SVneo cells via TEM. Furthermore, Met treatment was found to alleviate placental trophoblast injury more effectively. Both Met and the ferroptosis inhibitor deferoxamine (DFO) enhanced trophoblast proliferation, reduced iron levels and mitochondrial damage, and attenuated oxidative stress and inflammation. Western blotting and PCR analyses indicated that Met and DFO could reverse the expression of ferroptosis‐related factors in insulin resistance trophoblasts. Our study confirmed ferroptosis in GDM placental trophoblasts and showed that Met could more effectively mitigate placental trophoblast damage. Met may ameliorate GDM trophoblast injury by regulating ferroptosis via the NRF2/GPX4 pathway, which is linked to reducing oxidative stress and inflammation in GDM trophoblasts.

**Highlights**

1. Metformin (Met) alleviated ferroptosis damage (mitochondrial shrinkage and iron overload) in trophoblasts from GDM patients and insulin resistance (IR)–induced HTR‐8/SVneo cells.
2. In the cellular model, Met reduced iron content, reactive oxygen species (ROS), and inflammation, while increasing GSH levels and promoting cell proliferation.
3. Met upregulated ferroptosis inhibitors (NRF2 and glutathione Peroxidase 4 [GPX4]) and downregulated promoters (ATF2 and ACSL4) in IR trophoblasts.
4. Its protective effects matched those of the ferroptosis inhibitor deferoxamine (DFO) but not of an apoptosis inhibitor.
5. Met may protect gestational diabetes mellitus (GDM) trophoblasts by suppressing ferroptosis via the NRF2/GPX4 pathway.

## 1. Introduction

GDM is a common endocrine disorder in pregnancy, characterized by varying degrees of glucose metabolism abnormalities. The prevalence of GDM is gradually increasing due to rising obesity rates among women of childbearing age and advancing maternal age [1]. GDM poses significant short‐ and long‐term health risks to both the mother and the offspring. Its pathogenesis is influenced by multiple factors, including oxidative stress imbalance, chronic inflammation, dysregulated lipid metabolism, IR, and placental dysfunction [2]. GDM poses significant short‐ and long‐term health risks to both the mother and the offspring. Its pathogenesis is influenced by multiple factors, including oxidative stress imbalance, chronic inflammation, dysregulated lipid metabolism, IR, and placental dysfunction.

In recent studies, iron homeostatic imbalance has been identified as another risk factor for GDM. A large prospective cohort study in 2011 showed that increased intake of heme iron during the first trimester of pregnancy led to increased levels of oxidative stress in the body, thereby increasing the risk of GDM [3]. Zhu [4] et al. studied the association between iron supplement use and risk of GDM in 3289 pregnant women and confirmed that iron supplement use before pregnancy was associated with an increased risk of GDM (OR = 1.57, 95% CI [1.06–2.32]). This suggests that iron homeostasis imbalance plays an important role in GDM. Unstable iron generates ROS, which leads to oxidative stress and activates ferroptosis. Ferroptosis is a novel mode of programmed cell death identified in recent years, which is mainly characterized by iron overload, lipid peroxidation and disruption of the antioxidant system, especially depletion of GPX4 [5]. Serum ferritin levels are elevated in patients with iron overload, which is strongly associated not only with an increased risk of Type 2 diabetes but also with the development of GDM [6]. Therefore, targeting ferroptosis may be a potential treatment for GDM.

A total of 70%–85% of GDM patients can control their blood glucose only through lifestyle changes including healthy diet and appropriate physical exercise, but there are still some pregnant women with substandard blood glucose control who need to be treated with additional medication, of which insulin is the first choice of medication for the treatment of hyperglycemia in GDM [7]. However, in the last few years, Met as an alternative treatment to insulin has been included in the guidelines for GDM, even as first‐line treatment in some cases [8]. Compared with insulin alone, 46.3% of women in the Met treatment group needed insulin supplementation to achieve euglycemia, but the Met‐treated group had lower gestational weight gain, lower postprandial glycemia, fewer hypoglycemic events, and a lower cesarean section rate [9]. In a study of Met treatment for polycystic ovary syndrome (PCOS), it was found that Met treatment increased GPX4 expression in the ovaries of PCOS mice, thereby reversing the ferroptosis process [10]. Met inhibits chondrocyte ferroptosis by upregulating SLC7A11 and downregulating P53, delaying rat knee cartilage degeneration [11]. However, the relationship between Met and ferroptosis in GDM has not been reported.

Although existing studies have confirmed that ferroptosis plays a crucial role in the occurrence and development of GDM, it remains unclear whether Met regulates placental trophoblast injury by targeting ferroptosis. This study is aimed at exploring whether Met alleviates GDM‐associated trophoblast injury via the ferroptosis pathway, thereby providing theoretical and experimental evidence for Met in the treatment of GDM.

## 2. Materials and Methods

### 2.1. Tissue Samples

The subjects of this study were all Chinese. Singleton pregnant women (aged 22–39) who had regular obstetric checkups and delivered in Changzhou Maternal and Child Health Hospital from September 2020 to December 2022 were selected as the study subjects. Inclusion criteria were as follows: (i) permanent residents of Changzhou City, (ii) light physical labor, (iii) 75‐g oral glucose tolerance test (OGTT) at 24–28 weeks of pregnancy, and (iv) informed consent signed by pregnant women and their families. Exclusion criteria were as follows: (i) diabetes mellitus and hypertension before pregnancy; (ii) smoking, alcohol abuse, and so on during pregnancy; (iii) perinatal complications such as preeclampsia, pregnancy complicated with cholestasis, pregnancy complicated with infectious diseases, premature rupture of membranes, amniotic fluid meconium transfer to cesarean section, postpartum uterine infection, chromosomal abnormalities (quantitative and/or structural), and so on. The normal standards of 75 g OGTT are as follows: fasting blood glucose (FBG), 1‐h postprandial glucose (1‐h PG), 2‐h postprandial glucose (2‐h PG) are lower than 5.1, 10.0, 8.5 mmol/L, respectively [11]. Blood glucose value at any time point (mentioned above) reaches or exceeds the above standard; at the same time, FBG < 7.0 mmol/L and 2 − h PG < 11.1 mmol/L, GDM was diagnosed. The subjects diagnosed with GDM were first given diet control and blood glucose was monitored for 2 ~ 4 weeks. The criteria for good glycemic control were FBG < 5.3 mmol/L, 1‐h PG after meal < 7.8 mmol/L, and 2‐h PG after meal < 6.7 mmol/L. When blood glucose cannot be controlled satisfactorily by diet alone, insulin is the first‐line clinical treatment. However, some patients refuse insulin therapy due to psychological factors, poor compliance, or other reasons, and receive oral Met instead. A total of 150 participants were enrolled in this study, with 30 cases in each group. All groups were enrolled in a parallel and independent manner, and no 1:1 individual matching was performed. Participants were divided into five groups: the normal healthy pregnant women as the control group (Group N), the GDM group with satisfactory glycemic control after dietary intervention (Group G1), the GDM group with unsatisfactory glycemic control but refusal of medical treatment (Group G2), the oral Met treatment group (Group Met), and the insulin treatment group (Group Ins). Meanwhile, general clinical data of the five groups were collected, including maternal age, gravidity, parity, body weight, body mass index (BMI), neonatal birth weight, antenatal hemoglobin (Hb) (tested within 1 week before delivery), ferritin, and transferrin. Immediately after delivery, placental tissues were randomly collected from five sites on the maternal surface, 2 cm from the placental margin, avoiding infarcted, calcified, and vascular areas. Two slices were fixed in 10% formaldehyde solution and embedded in paraffin. Two slices were immersed in RNAlater preservation solution, placed in centrifuge tubes, numbered, and frozen at −80°C refrigerator. One slice was divided into 1 × 1 × 1 − mm^3^ tissues, freshly placed into precooled 2.5% glutaraldehyde fixative solution and stored in refrigerator at 4°C in the dark. This study was approved by the Medical Ethics Committee of Changzhou Maternal and Child Health Hospital (CZFY2022046). All participants were informed of the investigative nature of the study, and informed consent was obtained from them.

### 2.2. Main Reagents and Instruments

HTR‐8/SVneo cells, a trophoblast cell line, were used in the present study. Trophoblast cells are essential for placental formation and function, which play a pivotal role in the development of gestational diabetes. HTR‐8/SVneo cells (Cat. No. CL‐0765) were obtained from Wuhan Punosai Biotechnology Co., Ltd. RPMI‐1640 medium (Cat. No. C11875500BT) was purchased from Gibco. D‐glucose (Cat. No. G8270) was purchased from Sigma‐Aldrich. Insulin (Cat. No. HY‐P0035), Met (Cat. No. HY‐B0627), DFO (Cat. No. HY‐B0988), and Z‐VAD‐FMK (Cat. No. HY‐16658B) were purchased from MedChemExpress. The CCK‐8 kit (Cat. No. KGA317) was obtained from Jiangsu Kaiji Biologicals. Prussian blue staining kit (Cat. No. G1426), glucose detection kit (Cat. No. BC2500), ROS assay kit (Cat. No. CA1410), and glutathione (GSH) assay kit (Cat. No. BC1175) were all purchased from Beijing Solarbio Technology Company. The human insulin assay kit (Cat. No. ml064302) was purchased from mlbio Enzyme‐Linked Biology Company. Anti‐interleukin‐10 receptor (IL‐10R) antibody (Cat. No. 308811) and anti‐Tumor Necrosis Factor‐*α* (TNF‐*α*) antibody (Cat. No. 502908) were purchased from BioLegend, Inc. Primary antibodies against activating transcription factor‐2 (ATF2) (Cat. No. ab239361), Nuclear Factor‐Erythroid 2‐Related Factor 2 (NRF2) (Cat. No. ab62352), long‐chain Acyl‐CoA synthetase 4 (ACSL4) (Cat. No. ab155282), GPX4 (Cat. No. ab125066), and GAPDH (Cat. No. ab8245) were purchased from Abcam. Primers were synthesized by Shanghai Bioengineering Company, and reverse transcription and RT‐PCR kits were purchased from TaKaRa Company. The primers were purchased from Shanghai Bioengineering Company, and the reverse transcription and RT‐PCR kits (Cat. Nos. RR036B, RR086B) were purchased from TaKaRa Company.

### 2.3. Cell Modelling and Grouping

The cryopreserved HTR‐8/SVneo cells were thawed rapidly at room temperature and subsequently cultured in RPMI‐1640 medium supplemented with 10% fetal bovine serum and 100 U/mL penicillin–streptomycin. The cells were maintained in an incubator at 37°C with 5% CO_2_. To establish IR models, HTR‐8/SVneo cells were inoculated and treated for 72 h with RPMI‐1640 medium containing 25 mmol/L D‐glucose [12] and 10^−7^‐mmol/L insulin. The experimental groups were divided into control group (adding RPMI‐1640 medium), IR group (adding 25 mmol/L D‐glucose and 10^−7^ mol/L insulin), IR + Met group (IR + 0.5 mmol/L Met), IR + DFO group (IR + 50*μ*moL/L DFO) and IR + Z − VAD − FMK group (IR + 10*μ*mol/L Z − VAD − FMK) [13]. After the model was successfully established, cells were treated with the respective drugs for 48 h, then harvested for subsequent analyses.

### 2.4. CCK8 Detects Cell Proliferation

HTR‐8/SVneo cells from each group were seeded into 96‐well plates, followed by the addition of diluted CCK‐8 reagent. The absorbance at 450 nm was measured using a microplate reader, and cell viability was calculated accordingly.

### 2.5. Observation of Trophoblast Ultrastructure by Transmission Electron Microscope (TEM)

HTR‐8/SVneo cells or placental tissues were fixed in 2.5% glutaraldehyde and 1% osmium tetroxide at room temperature, dehydrated in a graded ethanol series, substituted with 100% acetone, embedded in resin, and sectioned. After double staining with 3% uranyl acetate and lead citrate, the sections were observed and photographed under TEM.

### 2.6. Determination of Biochemical Indexes in HTR‐8/SVneo

Glucose concentration was determined in cell supernatants according to the glucose assay kit instructions, with absorbance measured at 505 nm.

Homeostatic Model Assessment of Insulin Resistance (HOMA‐IR) detection were as follows: Supernatants of HTR‐8/SVneo cells were added to ELISA plates as required, followed by 100 *μ*L enzyme conjugate. After incubation and washing, chromogenic reagent was added for 15 min, the reaction was terminated with stop solution, and absorbance was measured at 450 nm. HOMA − IR = glucose content∗ insulin content/22.5.

For flow cytometry, cells were stained with PE antihuman TNF‐*α* or APC anti‐human CD210 (IL‐10R) antibody (5 *μ*g/test) at 37°C for 30 min in the dark, resuspended in PBS, and analyzed by flow cytometry.

### 2.7. ROS and GSH Determination

Intracellular ROS was detected using DCFH‐DA probe according to the manufacturer′s instructions. Cells were incubated with 10 *μ*mol/L DCFH‐DA at 37°C for 20 min in the dark, washed with PBS to remove excess probe, and DCF fluorescence was imaged under a fluorescence microscope.

GSH levels were determined by measuring absorbance at 412 nm in cell supernatants from the five groups, following the GSH assay kit protocol.

### 2.8. Determination of Cellular Iron Content

Ferrous iron levels were measured using a detection kit. After binding with the probe, absorbance was determined at 593 nm to calculate relative ferrous iron content. For Prussian blue staining, cells in 24‐well plates were fixed with 4% formaldehyde for 30 min, washed, incubated with Prussian blue working solution for 30 min, rinsed with distilled water, and examined under a microscope (200×).

### 2.9. Western Blot Experiments

Total protein was extracted from HTR‐8/SVneo cells, and concentration was measured. Proteins were separated by 10% SDS‐PAGE and transferred to PVDF membranes. After blocking with skimmed milk, membranes were incubated with primary antibodies overnight at 4°C, washed three times with PBST, and incubated with secondary antibodies for 1 h at room temperature. Following PBST washing, ECL luminescence was performed, and relative protein expression was analyzed using the Bio‐Rad Gel Doc EZ imaging system and ImageJ software.

### 2.10. RT‐qPCR Experiments

Total RNA was extracted from each group of cells using TRIzol reagent. After measuring the concentration and assessing the purity of the RNA, it was reverse‐transcribed into cDNA. Reverse transcription quantitative PCR (RT‐qPCR) was then performed using a real‐time PCR system with a SYBR Green kit. GAPDH served as the internal reference gene, and the relative gene expression was calculated using the 2^−*ΔΔ*Ct^ method. The primer sequences used are listed in Table 1.

**Table 1:** PCR primer pairs for each gene.

Gene	Primer sequences	Length (bp)
ATF2	Forward: TACAACAGCCAGCCACATCC	20
	Reverse: CTTCTCCGACGACCACTTGT	20
NRF2	Forward: TTCTTCGGCTACGTTTCAGTCA	22
	Reverse: CCGTCTAAATCAACAGGGGCT	21
GPX4	Forward: AGATCCAACCCAAGGGCAAG	20
	Reverse: AGGAACTGTGGAGAGACGGT	20
ACSL4	Forward: TGTGGTGCTGGGACAGTTAC	20
	Reverse: TCTGGGGTTTGGCTTGTCAT	20
GAPDH	Forward: AGATCATCAGCAATGCCTCCT	21
	Reverse: TGAGTCCTTCCACGATACCAA	21

### 2.11. Statistical Methods

Data were analyzed and graphed using GraphPad Prism 9.0 software and are presented as mean ± standard deviation (SD). Statistical analysis was based on comparisons of independent samples among multiple groups, and one‐way ANOVA was used for intergroup comparison, followed by the least significant difference (LSD) post hoc test for pairwise comparisons. A *p* value < 0.05 was considered statistically significant.

## 3. Results

### 3.1. Comparison of General Information and Pregnancy Outcomes

There was no significant difference in age, gestational week, gestational time and delivery time among the five groups (*p* > 0.05). The pre‐pregnancy BMI of the G1, Met, and Ins groups was higher than that of the N group (*p* < 0.05), the pre‐pregnancy BMI of the Ins group was higher than that of the G1 group (*p* < 0.05), and the pre‐pregnancy BMI of the Met and Ins groups was higher than that of the G2 group (*p* < 0.05). The weight of newborns in the N and G1 groups was lower than that in the G2 group (*p* < 0.05), and the number of cases of neonatal asphyxia in all five groups was 0. Prenatal Hb content in G1 group and Ins group was significantly higher than that in N group. Compared with INS group, the antenatal Hb content of MET group was decreased significantly. Compared with group N, ferritin content in Groups G1 and G2 was significantly increased. Transferrin content was increased in Groups G1 and G2, but not statistically significant. Both transferrin content and ferritin content decreased in group Met compared with Groups G1 and G2, but there was no statistical significance, which may be related to the small sample size (Table 2). After adjustment for confounding factors such as pre‐pregnancy BMI, multivariate linear regression analyses demonstrated no significant independent associations of maternal age, pre‐pregnancy BMI, gestational weight gain and gravidity with antenatal Hb levels (all *p* > 0.05) (Table S1). Additionally, neither of the multivariate linear regression models using ferritin or transferrin as the dependent variable reached statistical significance (see Supporting Information for details (available here)).

**Table 2:** Comparison of clinical data of pregnant women in five groups.

Variables	N group	G1 group	G2 group	Met group	Ins group	*F*	*p*
Age (year)	30.47 ± 4.05	33.03 ± 3.97	32.40 ± 4.52	32.30 ± 3.62	31.17 ± 4.20	1.926	0.109
Gestational age (weeks)	38.80 ± 0.67	38.73 ± 0.57	38.65 ± 0.73	39.06 ± 0.82	38.68 ± 0.58	1.751	0.142
Gravidity	2.43 ± 1.10	2.47 ± 1.20	2.77 ± 1.28	2.20 ± 1.03	2.53 ± 1.38	0.857	0.492
Parity	1.77 ± 0.68	1.63 ± 0.56	1.80 ± 0.61	1.47 ± 0.63	1.53 ± 0.78	1.456	0.219
BMI before pregnancy (kg/m^2^)	22.05 ± 2.20	23.58 ± 3.72^∗^	23.11 ± 3.42	24.94 ± 2.88^∗^^△^	25.26 ± 2.22^∗^^#△^	6.050	0.000
Weight gain during pregnancy (kg)	13.95 ± 2.98	12.33 ± 5.18	15.07 ± 5.87	12.16 ± 4.97	12.01 ± 3.03	2.761^a^	0.034
Newborn birth weight (g)	3353.33 ± 254.60^△^	3373.67 ± 157.67^△^	3653.67 ± 386.67	3634.67 ± 550.52	3496.33 ± 492.62	5.827^a^	0.000
Antenatal hemoglobin (g/L)	115.20 ± 9.47^▽^	122.97 ± 9.92^∗^	123.27 ± 9.35	119.20 ± 6.47^▽^	125.67 ± 7.64^∗^	6.721	0.000
Transferrin (g/L)	3.38 ± 0.39	3.42 ± 0.61	3.49 ± 0.41	3.27 ± 0.37	3.36 ± 039	1.068	0.375
Ferritin	10.51 ± 3.23	15.41 ± 11.93^∗^	16.17 ± 6.85^∗^	12.64 ± 2.94	15.24 ± 6.92^∗^	3.248^a^	0.014
Asphyxia neonatorum cases	0	0	0	0	0	_	_

*Note:* Compared with N group,  ^∗^ *p* < 0.05; compared with G1 group, ^#^ *p* < 0.05; compare with Ins group, ^▽^ *p* < 0.05; compared with G2 group, ^△^ *p* < 0.05. *n* = 30 for each group.

^a^Welch method.

### 3.2. Ultrastructure and Iron Deposition of Placenta

**Figure 1:**
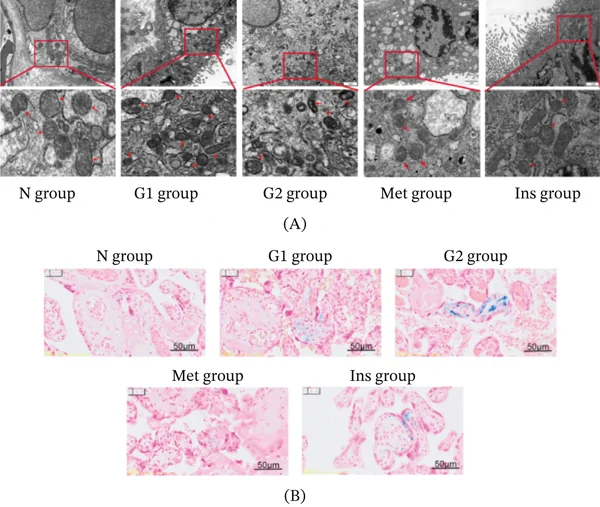
Ultrastructure and iron deposition of placenta. (A) Ultrastructure of placental trophoblast in five groups under TEM and (B) Prussian blue staining for detecting iron deposition in the placenta (×200).

Under the field of view of TEM, the mitochondrial structure of placental trophoblast cells in the N group was essentially normal. However, in the GDM placental trophoblast cells of the G1, G2, Met, and Ins groups, phenomena such as mitochondrial atrophy, reduction in size, increased mitochondrial membrane density, rounding of mitochondria, and deepening of mitochondrial cristae were observed. Among these, the G2 group showed the most severe changes, highly consistent with the characteristics of ferroptotic cells. These data indicate that both Met and insulin can ameliorate mitochondrial damage, with Met showing a more significant improvement (Figure 1A).

In the Prussian blue staining experiment, iron is stained blue, the cytoplasm appears light pink, and the nucleus is stained red. After staining, iron‐containing particles were visible in placental tissues from all five groups. Compared with the N group, the GDM placentas in the G1, G2, Met, and Ins groups showed a significant increase in iron‐containing particles. Compared with the G2 group, the Met and Ins groups had a noticeable decrease in iron‐containing particles in the placenta (Figure 1B).

### 3.3. Expression Changes of Ferroptosis‐Related Marker Genes in the Placenta

**Figure 2:**
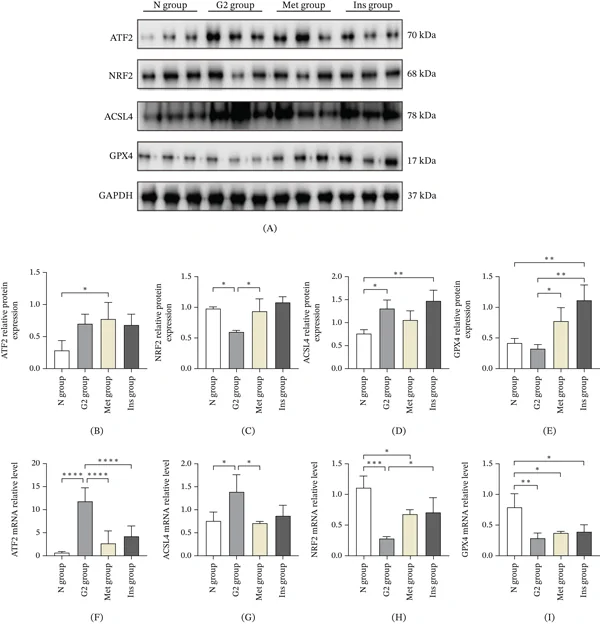
Expression changes of ferroptosis‐related marker genes in the placenta. (A–E) The relative expression of ATF2, NRF2, ACSL4, and GPX4 in placenta of N group, G2 group, Met group and Ins group were detected by (A) Western blot and (B–E) their bar graphs were also presented; (F–I) mRNA level of ATF2, NRF2, ACSL4 and GPX4 in placenta of *N* group, G2 group, Met group, and Ins group. (N group represents normal pregnant women [without pregnancy complications], G2 group represents pregnant women with poor glycemic control after diet control, Met group represents pregnant women with metformin alone, and Ins group represents pregnant women with insulin alone).

Compared with the N group, the expression of NRF2 protein in the G2 group was significantly decreased, and the expression of ACSL4 protein was significantly increased (*p* < 0.05), and the mRNA expression of ATF2 and ACSL4 was significantly increased (*p* < 0.05). Compared with the G2 group, the expression of NRF2 and GPX4 proteins in the Met group was significantly increased. The expression of ATF2 and ACSL4 mRNA was significantly reduced. In the Ins group, the content of GPX4 protein was also higher than that in the G2 group (*p* < 0.05) (Figure 2).

### 3.4. Met Can Improve the Proliferation of IR Cells

**Figure 3:**
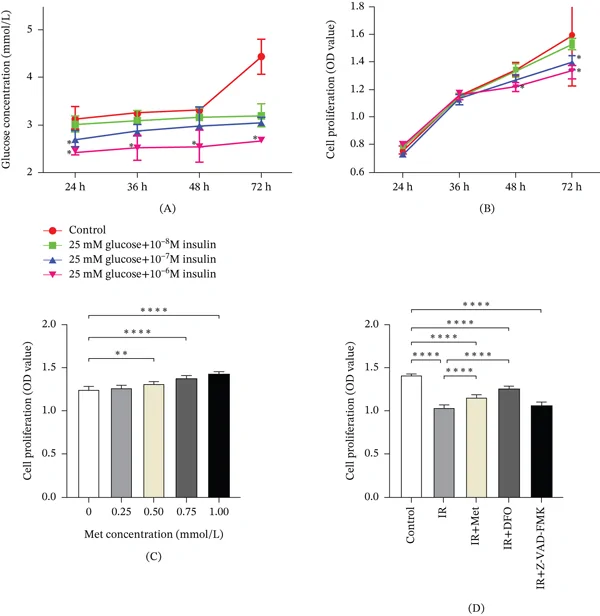
Metformin can improve the proliferation of IR cells. (A) Glucose concentration of HTR‐8/SVneo cells treated with 10^−8^ ~ 10^−6^ mol/L insulin for 24, 36, 48, and 72 h in 25 mmol/L glucose environment; (B) proliferation ability of HTR‐8/SVneo cells treated with 10^−8^ ~ 10^−6^ mol/L insulin for 24, 36, 48, and 72 h in high glucose environment; (C) proliferation ability of IR cells treated with different metformin for 48 h; D, Proliferation ability of HTR‐8/SVneo cells treated with different drugs.

Under high glucose conditions (25‐mmol/L glucose), HTR‐8/SVneo cells treated with 10^−7^‐mol/L insulin showed a significantly enhanced ability to consume glucose over 24 h, with a statistically significant difference (*p* < 0.05). In the insulin‐treated group, glucose consumption tended to stabilize after 36 h, with no significant difference compared with the control group (*p* > 0.05), suggesting the development of IR (Figure 3A). Under high glucose conditions (25 mmol/L glucose), HTR‐8/SVneo cells treated with a concentration of 10^−7^ mol/L insulin for 72 h showed a significant decrease in cell viability (*p* < 0.05) (Figure 3B). It was ultimately determined that treating cells with 10^−7^ mol/L insulin for 72 h under the condition of 25‐mmol/L glucose can successfully induce an IR model. Compared with the control group (the concentration of Met is 0 mmol/L), the proliferative capacity of HTR‐8/SVneo cells treated with 0.5, 0.75, and 1‐mmol/L Met was significantly increased (*p* < 0.05). Therefore, 0.5‐mmol/L Met was used to treat HTR‐8/SVneo cells in the subsequent experiments (Figure 3C). The proliferative capacity of cells in the IR group decreased. Compared with the IR group, the proliferative capacity of trophoblasts treated with Met and DFO was significantly improved (*p* < 0.05) (Figure 3D).

### 3.5. Met Reduces Iron Content and Mitigates Mitochondrial Damage

**Figure 4:**
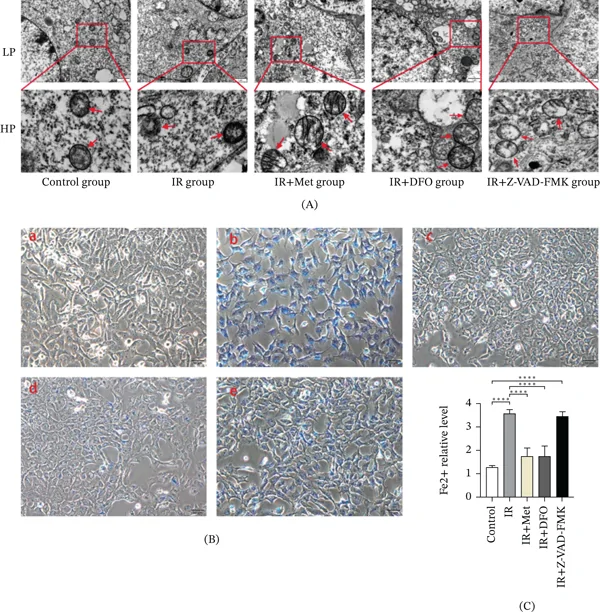
Metformin reduces iron content and mitigates mitochondrial damage. (A) Ultrastructure of HTR‐8/SVneo cells treated with different drugs under electron microscope; (B) Prussian blue staining of HTR‐8/SVneo cells in five groups (a–e indicate the control, IR, IR + Met, IR + DFO, and IR + Z − VAD − FMK groups, respectively). Iron stained blue, cytoplasm pale pink, nucleus red by Prussian blue staining; (C) Fe^2+^ contents of HTR‐8/SVneo cells in five groups (*F*(4, 10) = 47.474, *p* < 0.01, *η* ^2^ = 0.95). (compared with control group,  ^∗^ *p* < 0.05; compared with IR group, ^#^ *p* < 0.05).

To investigate the effect of Met on ferroptosis, we treated IR HTR‐8/SVneo trophoblasts with Met. For comparison, the iron chelator DFO was used, as it is known to reduce Fe^2+^ content, decrease substrates for the Fenton reaction, attenuate ROS generation, inhibit lipid peroxidation, and consequently suppress ferroptosis and mitigate ferroptosis‐related injury. To differentiate from apoptosis, we added the apoptosis inhibitor Z‐VAD‐FMK as a contrast. As shown by the red arrows in Figure 4A, compared with the control group, the mitochondria in the IR group exhibited characteristics of atrophy, reduced size, increased membrane density, rounding, and deepening of cristae. Compared with the IR group, treatment with Met and DFO improved the aforementioned mitochondrial damage phenotypes, whereas treatment with apoptosis inhibitors did not significantly correct the mitochondrial damage (Figure 4A).

Iron particles can be visualized as blue after Prussian blue staining. Compared with the control group, the IR model showed a significant increase in iron particles. Treatment with Met and DFO resulted in a noticeable decrease in iron particles and Fe^2+^ content compared with the IR group (*p* < 0.05), whereas treatment with Z‐VAD‐FMK did not result in any significant changes in iron particles and Fe^2+^ content (Figure 4B,C). However, no quantitative analysis was performed on Prussian blue staining in this study, which constitutes a certain limitation.

### 3.6. Met Treatment Reduces the Inflammatory Response and Oxidative Stress Levels in Trophoblast Cells

**Figure 5:**
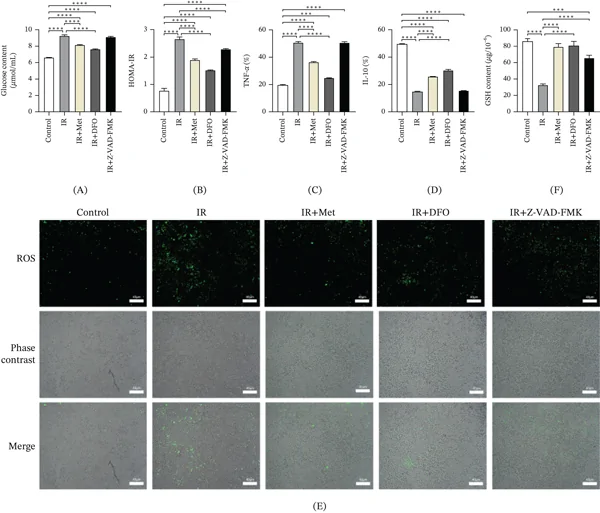
Met treatment reduces the inflammatory response and oxidative stress levels in trophoblast cells. (A) Glucose content of HTR‐8/SVneo cells in the control group, IR group, IR + Met group, IR + DFO group, and IR + Z − VAD − FMK group (*F*(4, 10) = 380.536, *p* < 0.01, *η* ^2^ = 0.993); (B) insulin resistance index of HTR‐8/SVneo cells in the control group, IR group (*F*(4, 10) = 479.413, *p* < 0.01, *η* ^2^ = 0.995), IR + Met group, IR + DFO group, and IR + Z − VAD − FMK group; (C) TNF‐*α* level of HTR‐8/SVneo cells in the control group, IR group, IR + Met group, IR + DFO group, and IR + Z − VAD − FMK group (*F*(4, 10) = 583.371, *p* < 0.01, *η* ^2^ = 0.996); (D) IL‐10 level of HTR‐8/SVneo cells in the control group, IR group, IR + Met group, IR + DFO group, and IR + Z − VAD − FMK group (*F*(4, 10) = 1805.756, *p* < 0.01, *η* ^2^ = 0.999); (E) DCFH‐DA fluorescent probe staining, ROS will be dyed green; (F) GSH content of HTR‐8/SVneo cells in the control group, IR group, IR + Met group, IR + DFO group, and IR + Z − VAD − FMK group (*F*(4, 10) = 89.965, *p* < 0.01, *η* ^2^ = 0.973). ( ^∗^ *p* < 0.05,  ^∗∗^ *p* < 0.01,  ^∗∗∗^ *p* < 0.001,  ^∗∗∗∗^ *p* < 0.0001).

Both the glucose concentration and HOMA‐IR in the IR group were significantly higher than those in the control group (*p* < 0.05).the glucose concentration was reduced, and the IR of trophoblast cells was improved, with no significant statistical difference between the DFO group and the MET group (Figure 5A,B). Similarly, compared with the control group, the proinflammatory cytokine TNF‐*α* was elevated, and the anti‐inflammatory cytokine IL‐10 was reduced in the IR group (*p* < 0.05). These conditions were improved after treatment with Met and DFO, but treatment with apoptosis inhibitors did not significantly improve the aforementioned phenomena (Figure 5C,D).

In the IR group, the level of ROS fluorescence was significantly higher than that of the control group, whereas the content of GSH was lower than that of the control group. Compared with the IR group, the ROS fluorescence level decreased and the content of GSH increased after treatment with Met and DFO, but the treatment with the apoptosis inhibitor did not significantly improve these phenomena (Figure 5E,F).

### 3.7. Met Alters Ferroptosis‐Related Genes

**Figure 6:**
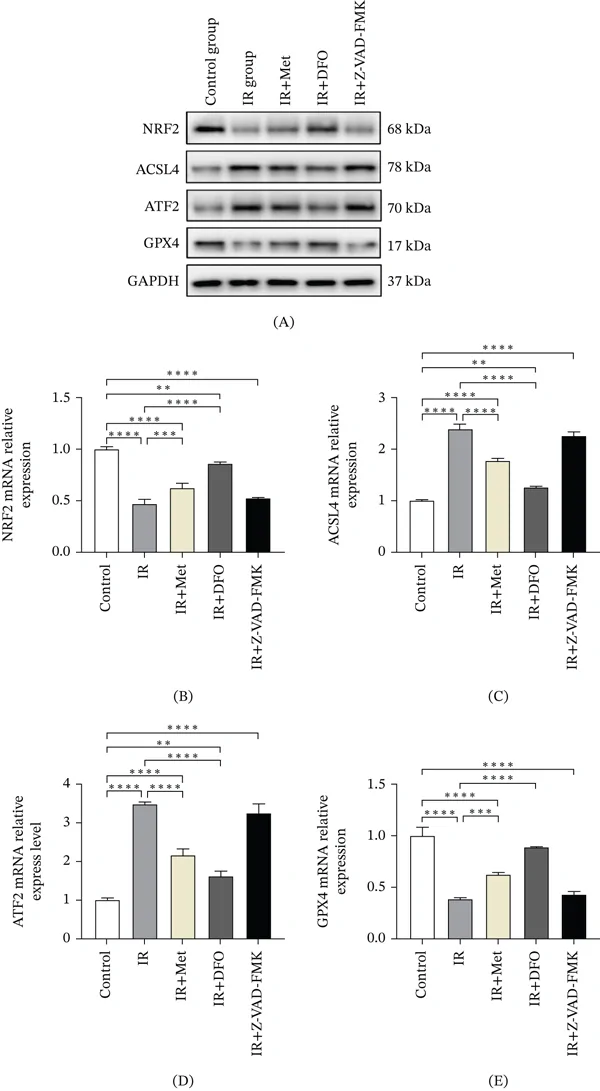
Metformin alters ferroptosis‐related genes. (A) The expression of NRF2, ACSL4, ATF2 and GPX4 in five groups were detected by Western blot (A); (B–E) relative expression level of ferroptosis‐related gene mRNA in five groups of cells. Control group (adding RPMI‐1640 medium), IR group (adding 25 mmol/L D‐glucose and 10^−7^ mol/L insulin), IR + Met group (IR + 0.5 mmol/L Met), IR + DFO group (IR + 50 *μ*mol/L DFO), and IR + Z − VAD − FMK group (IR + 10 *μ*mol/L Z − VAD − FMK). ( ^∗^ *p* < 0.05,  ^∗∗^ *p* < 0.01,  ^∗∗∗^ *p* < 0.001,  ^∗∗∗∗^ *p* < 0.0001).

This experiment examined the expression of key genes related to ferroptosis, including inhibitors (NRF2 and GPX4) and promoters (ACSL4 and ATF2) of ferroptosis. The experimental data showed that in the IR group, the expression of ACSL4 and ATF2 increased, whereas the expression levels of NRF2 and GPX4 significantly decreased. Treatment with Met and DFO could reverse the expression levels of ferroptosis‐related genes. Unlike Met and DFO, treatment with apoptosis inhibitors did not result in significant changes in ferroptosis‐related genes (*p* > 0.05) (Figure 6). This indicates that Met can regulate the expression of genes related to ferroptosis.

## 4. Discussion

Morphological characteristic changes of ferroptosis in trophoblast cells from GDM. Ferroptosis has been a research hotspot in the past decade, and numerous studies have confirmed its involvement in the development of various diseases, including neurodegenerative diseases (Alzheimer′s disease, etc.) [14], cardiovascular disease, tumors, brain damage, diabetes, pregnancy‐related diseases (premature birth, fetal growth restriction, and preeclampsia) [15]. Ferroptosis, distinct from autophagy and apoptosis, exhibits unique morphological changes. The main manifestations include shrunken mitochondria, a reduction or even disappearance of mitochondrial cristae, increased mitochondrial membrane density, smaller mitochondria, and a normal cell nucleus morphology, but lacking chromatin condensation [16]. In this study, it was observed that trophoblast cells from GDM placentas in all four groups exhibited varying degrees of ferroptosis, with the most pronounced mitochondrial changes in the poorly glycemic‐controlled G2 group. Both Met and insulin can ameliorate mitochondrial damage, with Met showing a more significant improvement. In the cellular model, trophoblast cells from four groups of IR placentas all exhibited morphological changes of ferroptosis to varying degrees. Compared with Z‐VAD‐FMK, the improvement of mitochondrial damage after Met and DFO treatment was more significant. This suggests that Met, like DFO, significantly ameliorates ferroptosis in IR trophoblast cells.

A meta‐analysis involving 4690 pregnant women revealed a positive correlation between ferritin levels and the risk of GDM. Compared with pregnant women with the lowest ferritin levels, those with the highest ferritin levels had an 87% increased prevalence of GDM (RR: 1.87, 95% CI [1.50–2.34]) [17]. A large prospective cohort study conducted by Bowers demonstrated that after adjusting for other dietary and nondietary risk factors for GDM, long‐term excessive intake of heme iron was more likely to result in higher iron storage in the body, thereby increasing the risk of GDM [18]. This phenomenon further confirms that abnormal iron metabolism is closely associated with the pathogenesis of GDM. After multivariate adjustment, no significant differences in serum ferritin and transferrin levels were observed among all groups in clinical samples, which may be attributed to the relatively limited sample size in this study. Considering the close relationship between ferroptosis and excessive iron load, this experiment directly examined the deposition of iron particles and the content of Fe^2+^. The results showed that normal placentas contained very few iron particles, whereas the G2 group, which had inadequate glycemic control, had the highest amount of iron particles. In the four IR groups of trophoblast cells, the iron content was significantly higher than that in the control group, while the addition of Met and DFO significantly reduced the iron content compared with the IR group. This study confirmed the presence of ferroptosis in trophoblast cells from GDM patients, and ferroptosis is related to the severity of the patient′s condition and the medication used for treatment. Treatment with Met and insulin can both improve ferroptosis in GDM trophoblast cells, which is consistent with Han et al.′s results [19]. Furthermore, Met has a more significant effect in reducing iron content and improving mitochondrial damage compared with insulin.

### 4.1. Effect of Met on Ferroptosis in GDM

Met, as an oral therapeutic agent for GDM, is used in clinical work to control blood glucose in pregnant women who are unable to use insulin for subjective or objective reasons, or when glycemic control is poor with insulin alone [7]. The main hypoglycemic mechanism of Met is the inhibition of hepatic gluconeogenesis and glycogenolysis via the AMPK pathway [20]. Meanwhile, Met has been found to inhibit the inflammatory response and ferroptosis of vascular cells through the Adenosine 5 ^′^‐monophosphate‐activated protein kinase (AMPK)/extracellular regulated protein kinases (ERK) pathway, thereby attenuating the progression of atherosclerosis [21]. Met can regulate ferroptosis through the Sirtuin 3 (SIRT3)/AMPK/mammalian target of rapamycin (mTOR) pathway to improve body ovarian dysfunction in PCOS mice [19]. This indirectly suggests that the ferroptosis pathway may be inhibited when Met exerts its hypoglycemic effect via the AMPK pathway. Met has also been shown to protect pancreatic *β*‐cells by regulating GPX4/ACSL4 axis to alleviate glycolipid‐induced ferroptosis in pancreatic beta cells [22]. Available studies have focused on the role of Met on ferroptosis in other diseases, but the role of Met with ferroptosis in GDM cells has not been elucidated.

In this experiment, IR trophoblasts were treated with Met, the apoptosis inhibitor Z‐VAD‐FMK and the ferroptosis inhibitor DFO, respectively. The proliferation ability of IR trophoblasts was lower than that of normal trophoblasts. The proliferation ability of cells in Met and DFO groups was significantly improved compared with IR group. At the same time, glucose content and HOMA‐IR in cells decreased significantly compared with IR group. This suggested that Met improves the proliferation, glucose tolerance and IR similar to the ferroptosis inhibitor DFO, so we can hypothesize that Met may improve GDM cell injury by inhibiting ferroptosis‐related pathways.

Met regulates the expression of ferroptosis‐related genes. Currently, ferroptosis is mainly regulated through three pathways: (1) Ferric ion accumulation and iron homeostasis regulation, NRF2 is a key regulator of the endogenous antioxidant pathway, activation of the Nrf2/heme oxygenase 1 (HO‐1) pathway can reduce ferric ion accumulation, inhibit ferroptosis, and ameliorate drug‐induced liver injury [23]. (2) Antioxidant System GSH/GPX4 pathway: GPX4 as an antioxidant enzyme is a key upstream regulator of ferroptosis. The ferroptosis inducer erastin activates the cystine/glutamate reverse transport system, leading to GSH depletion and subsequent GPX4 inactivation [24]. (3) Lipid metabolism pathway, which is polyunsaturated fatty acids, are prone to oxidation, and ACSL4 is involved in activating and promoting their incorporation into phospholipids and driving the subsequent onset of cellular ferroptosis [25]. In addition to the classical pathways of occurrence mentioned above, ATF2 is a key gene promoter of inflammatory signalling [26], which was found to be highly expressed in GDM placentas in our previous study. In this study, compared with control group, ATF2 and ACSL4 expression levels increased, NRF2 and GPX4 expression decreased in G2 and IR groups. Compared with G2 group, Met treatment significantly reversed the expression levels of NRF2 and GPX4, but no significant differences were seen for ATF2 and ACSL4 proteins. The relative expression levels of GPX4 and NRF2 mRNA were significantly increased and ATF2 and ACSL4 mRNA expression were significantly decreased by the addition of Met to IR trophoblast cells. In nonalcoholic fatty liver disease (NAFLD) rats, Met can exert a protective effect on rat liver by activating the Nrf2/Gpx4 pathway, inhibiting ferroptosis, and attenuating lipid peroxidation [27]. Nrf2/Gpx4 pathway activation also ameliorates high‐fat diet‐induced IR [28]. Therefore, we hypothesized that Met may exert an inhibitory effect on ferroptosis through NRF2/GPX4 pathway activation. This experiment demonstrates the therapeutic advantage of Met in GDM in terms of ameliorating ferroptosis.

Met ameliorates IR trophoblast injury, which may be related to oxidative stress and inflammation. Ferroptosis is mainly caused by iron overload that leads to the Fenton reaction, lipid ROS accumulation, imbalance of intracellular oxidative and reductive systems, reduced antioxidant capacity, leading to mitochondrial damage and disruption of cellular membrane integrity, which ultimately causes oxidative cell death [29]. ROS are oxygen radicals and nonradical derivatives, which are important by‐products of oxidative metabolism and play an important role in cellular activities. As a substrate of GPX4, GSH not only has antioxidant effects but also participates in ferroptosis [30]. Therefore, the decrease of antioxidant GSH and the increase of lipid ROS may be important markers of ferroptosis. Oxidative stress has been shown to play an important role in GDM. The pregnant women with GDM have elevated levels of oxidative stress and impaired free radical elimination mechanisms compared with normal pregnancies [31]. In our study, ROS levels were significantly increased and GSH levels significantly decreased in the IR group. After Met treatment, ROS fluorescence level decreased, whereas GSH level increased significantly. The effect of Met was consistent with that of DFO. Met attenuated Fe^2+^ contents, lipid peroxidation, and ROS accumulation, and increased the antioxidant factor GSH content, which are the main factors of ferroptosis, so we believe that Met can improve the level of ferroptosis‐associated oxidative stress and thus improve cell damage.

In a cohort study, women with GDM exhibited elevated serum inflammatory cytokine levels during pregnancy, suggesting inflammation may be involved in the pathogenesis of GDM [32]. Elevated concentrations of pro‐inflammatory cytokines, particularly plasma TNF‐*α*, are strongly associated with abnormal glucose metabolism and IR in patients with GDM [33, 34]. Desoye Gfound that TNF‐*α* down‐regulates insulin action, leading to IR during pregnancy [35]. Ferroptosis, as a new mode of cell death, is also closely related to inflammation. When ferroptosis occurs, TNF‐*α* levels in tissues are significantly elevated, and the application of ferroptosis inhibitors can reduce TNF‐*α* levels [36]. The anti‐inflammatory factor IL‐10 plays a role in reducing neuroinflammation and promoting hematoma clearance, significantly reducing bleeding‐induced lipid ROS accumulation and subsequent neuronal ferroptosis [37]. Chen suggested that ferroptosis in GDM placental macrophages was significantly higher than in normal placenta, which may lead to the secretion of inflammatory cytokines involved in the inflammatory response by GDM placental tissues [38]. In our study, the level of the inflammatory factor TNF‐*α* was significantly higher and the anti‐inflammatory factor IL‐10 was significantly lower in IR trophoblasts compared with controls. Met treatment, like DFO, reversed this change in inflammatory factor levels. Therefore, we hypothesized that Met could improve ferroptosis‐related inflammatory indices thereby ameliorating cellular damage.

This study demonstrated the presence of ferroptosis in GDM trophoblasts at the human placental and cellular levels, and that ferroptosis was correlated with the severity of the patient′s disease and the patient′s therapeutic medication. The treatment with both Met and insulin improved the ferroptosis in GDM placental trophoblasts. Distinct from apoptosis inhibitors, Met, like DFO, can mediate ferroptosis and attenuate IR‐induced trophoblast damage, providing a theoretical basis for Met treatment of GDM. Met may modulate IR‐induced ferroptosis in trophoblast cells through the NRF2/GPX4 pathway, a process associated with improved inflammatory response and oxidative stress levels.

In summary, ferroptosis is involved in GDM trophoblast injury and may be one of the pathogenic mechanisms of GDM. Met may ameliorate GDM trophoblast injury by regulating ferroptosis through the NRF2/GPX4 pathway, a process that is associated with improved oxidative stress and inflammatory response in GDM trophoblasts. The iron ion chelator DFO reduced Fe^2+^ levels and improved trophoblast ferroptosis injury. Since ferroptosis is associated with iron overload, maternal iron supplementation should be controlled during pregnancy [39].

However, this study still has several limitations. In clinical practice, the number of GDM pregnant women who have indications for hypoglycemic medication but refuse treatment and consequently present with poor glycemic control is relatively small. The sample size was determined based on previous similar studies and clinical feasibility, and thus the sample size was not sufficiently large. Furthermore, since glycemic control status, treatment regimens, therapeutic indications, and medication adherence of pregnant women in each group were determined according to routine clinical practice rather than random allocation, and gestational iron supplementation was not adjusted for in the analysis, potential imbalances in baseline characteristics may exist across groups, which constitutes an inherent limitation of this study. In addition, the GDM cell model was established by inducing IR in vitro, which has certain limitations. Finally, this study lacks verification by animal experiments and in‐depth mechanistic research on the regulation of ferroptosis by Met.

**Nomenclature**
GDM: gestational diabetes mellitus
Met: metformin
IR: insulin resistance
HOMA‐IR: Homeostatic Model Assessment for Insulin Resistance
ROS: reactive oxygen species
GSH: reduced glutathione
IL‐10: interleukin‐10
TNF‐ɑ: tumor necrosis factor‐*α*
ATF2: activating transcription factor‐2
ACSL4: long‐chain acyl‐CoA synthetase‐4
GPX4: glutathione peroxidase‐4
NRF2: nuclear factor‐erythroid 2‐related Factor 2
DFO: deferoxamine
PCOS: polycystic ovary syndrome
FBG: fasting blood glucose
PG: postprandial glucose
Ins: insulin
BMI: body mass index
AMPK: Adenosine 5 ^′^‐monophosphate‐activated protein kinase
ERK: extracellular regulated protein kinases
SIRT3: Sirtuin 3
mTOR: mammalian target of rapamycin
HO‐1: heme Oxygenase 1
NAFLD: nonalcoholic fatty liver disease
OGTT: oral glucose tolerance test
CCK‐8: Cell Counting Kit‐8

## Author Contributions

Data curation: Q.L., X.Q., M.S., D.X., X.C., G.S., H.W., and W.Z.; formal analysis: Q.L., X.Q., M.S., D.X., and X.C.; investigation: Q.L., X.Q., and M.S.; methodology: Q.L., X.Q., and M.S.; project administration: Q.L., H.W., and W.Z.; resources: D.X. and X.C.; software: G.S.; supervision: H.W. and W.Z.; writing—original draft: Q.L. and X.Q.; writing—review and editing: H.W. and W.Z. Q.L. and X.Q. have contributed to the work equally and should be regarded as co‐first authors.

## Funding

This study was supported by the grants from the Applied Basic Research Project of Changzhou Science and Technology Bureau (No. CJ20220117) and High‐Level Leading Talents Project of Changzhou Health Commission (No. 2022CZLJ023).

## Ethics Statement

This study was approved by the Medical Ethics Committee of Changzhou Maternal and Child Health Hospital (CZFY2022046). All participants were informed of the investigative nature of the study and informed consent was obtained from them. This study also adhered to the Declaration of Helsinki.

## Consent

The authors have nothing to report.

## Conflicts of Interest

The authors declare no conflicts of interest.

## Supporting information


**Supporting Information** Additional supporting information can be found online in the Supporting Information section. Table S1: Factors associated with prenatal Hb by multiple linear regression.

## Data Availability

Data are available on request from the authors.
