# Supplementary material for: Metformin and Placental Ferroptosis in Gestational Diabetes: A Mechanistic Study
Source: J Diabetes Res. 2026 Jun 3;2026:2226729. doi: 10.1155/jdr/2226729 (PMC13581051; doi:10.1155/jdr/2226729)
Supplement: Supplementary file 1 [file JDR-2026-2226729-s001.docx]

Table S1: Factors associated with prenatal Hb by multiple linear regression

|  | B | SE | β | t | *P* | VIF |
| --- | --- | --- | --- | --- | --- | --- |
| Variable | 219.997 | 42.135 |  | 5.221 | <0.01^**^ |  |
| Age (year) | 0.293 | 0.18 | 0.13 | 1.629 | 0.106 | 1.164 |
| Gestational age (weeks) | -2.629 | 1.047 | -0.194 | -2.512 | 0.013^*^ | 1.093 |
| BMI before pregnancy (kg/m2) | -0.388 | 0.239 | -0.131 | -1.626 | 0.106 | 1.19 |
| Weight gain during pregnancy (kg) | -0.085 | 0.156 | -0.042 | -0.545 | 0.586 | 1.107 |
| **Group (reference = N group)** | | | | | | |
| G1 Group | 7.306 | 2.259 | 0.315 | 3.234 | 0.002 | 1.734 |
| G2 Group | 8.987 | 2.34 | 0.387 | 3.841 | <0.01^**^ | 1.86 |
| MET Group | 4.635 | 2.323 | 0.2 | 1.995 | 0.048 | 1.834 |
| INS Group | 10.275 | 2.232 | 0.443 | 4.603 | <0.01^**^ | 1.693 |
| **Group (reference = G1 group)** | | | | | | |
| N Group | -7.306 | 2.259 | -0.315 | -3.234 | 0.002^*^ | 1.734 |
| G2 Group | 1.681 | 2.221 | 0.072 | 0.757 | 0.45 | 1.676 |
| MET Group | -2.67 | 2.229 | -0.115 | -1.198 | 0.233 | 1.688 |
| INS Group | 2.969 | 2.222 | 0.128 | 1.336 | 0.184 | 1.678 |
| **Group (reference = G2 group)** | | | | | | |
| N Group | -8.987 | 2.34 | -0.387 | -3.841 | <0.001^***^ | 1.86 |
| G1 Group | -1.681 | 2.221 | -0.072 | -0.757 | 0.45 | 1.676 |
| MET Group | -4.352 | 2.227 | -0.188 | -1.954 | 0.053 | 1.686 |
| INS Group | 1.288 | 2.305 | 0.056 | 0.559 | 0.577 | 1.806 |
| **Group (reference = MET group)** | | | | | |  |
| N Group | -4.635 | 2.323 | -0.2 | -1.995 | 0.048 | 1.834 |
| G1 Group | 2.67 | 2.229 | 0.115 | 1.198 | 0.233 | 1.688 |
| G2 Group | 4.352 | 2.227 | 0.188 | 1.954 | 0.053 | 1.686 |
| INS Group | 5.64 | 2.287 | 0.243 | 2.466 | 0.015^*^ | 1.777 |
| Model Summary：R=.484a，R^2=^0.235，Adj.R^2^=0.185，F=4.768.P<0.001,D-W=2.105 | | | | | | |

(**P*<0.05, ***P*<0.01, ****P*<0.001, *****P*<0.0001)

As the linear association between pre-pregnancy BMI and prenatal hemoglobin was not satisfied, the assumption for analysis of covariance (ANCOVA) was violated.**Multiple linear regression** was performed with group as dummy variables (group N as reference), adjusted for age, gestational age, BMI, weight gain, gravidity, parity, and neonatal birth weight.A two-sided P < 0.05 was considered statistically significant. After adjustment for age, gestational age, pre-pregnancy BMI, weight gain, and gravidity, multiple linear regression showed that prenatal HB levels were significantly higher in the G1, G2, MET, and INS groups than in the N group (B=7.306, P=0.002; B=8.987, P＜0.001; B=4.635, P=0.048; B=10.275, *P*＜0.001).In addition, gestational age may have an independent effect on prenatal Hb level (B=-2.629, *P*=0.013).

Multiple linear regression analysis was performed with ferritin as the dependent variable. After adjusting for age, gestational age, pre-pregnancy BMI, gestational weight gain, and gravidity, the overall regression model was not statistically significant (F=1.846, *P*=0.065). No independent difference in ferritin levels was found among the groups.

Similarly, after adjusting for age, gestational age, pre-pregnancy BMI, gestational weight gain, and gravidity, the multiple linear regression model with **transferrin** as the dependent variable was not statistically significant (F=0.823, *P*=0.065).
